# Arabidopsis ubiquitin-conjugating enzyme UBC22 is required for female gametophyte development and likely involved in Lys11-linked ubiquitination

**DOI:** 10.1093/jxb/erw142

**Published:** 2016-04-10

**Authors:** Sheng Wang, Ling Cao, Hong Wang

**Affiliations:** ^1^Department of Biochemistry, University of Saskatchewan, Saskatoon, SK S7N 5E5, Canada; ^2^National Key Laboratory of Crop Genetic Improvement, College of Plant Science and Technology, Huazhong Agricultural University, Wuhan 430070, China

**Keywords:** Arabidopsis, female gametophyte, functional megaspore, gametogenesis, protein ubiquitination, ubiquitin-conjugating enzyme, UBC22.

## Abstract

Protein ubiquitination is critical for numerous processes in eukaryotes. The ubiquitin-conjugating enzyme (E2) is required for ubiquitination. The Arabidopsis genome has approximately 37 E2 genes, but *in vivo* functions for most of them remain unknown. In this study we observed that knockout mutants of Arabidopsis *UBC22* had much-reduced silique length and seed number, with nearly 90% of ovules aborted. Analyses revealed that the majority of mutant embryo sacs displayed severe defects and often contained no gamete nuclei. There was no difference between mutant and wild-type Arabidopsis at the megaspore mother cell stage; however, the functional megaspore was either not present or appeared abnormal in a large portion of mutant ovules, suggesting that the defect started with functional megaspore degeneration in the mutants. Degeneration continued during megagametogenesis, such that the percentage of mature embryo sacs without any gamete nuclei was much greater than the percentage of developing ovules without a functional megaspore and, in addition, various abnormalities in megagametogenesis were observed. Additionally, heterozygous plants had only 13.1% of ovules aborted, indicating that the heterozygous sporophytic tissues could affect the development of the mutant female gametophyte. UBC22 is the sole member of an Arabidopsis E2 subfamily, and is more closely related to one type of E2s in animals that catalyzes Lys11-specific ubiquitination. Indeed, our results showed that Arabidopsis UBC22 could catalyze ubiquitin dimer formation *in vitro* in a Lys11-dependent manner, suggesting that it likely catalyzes Lys11-linked ubiquitination in plants. This study has thus identified one biochemical property of UBC22 and revealed a novel function in female gametophyte development.

## Highlight

Arabidopsis UBC22 functions in the atypical Lys11-linked ubiquitination, which differs from the more common Lys48-linked ubiquitination, and its knockout results in degeneration of the functional megaspore and female gametes.

## Introduction

Protein ubiquitination, the process through which the small protein ubiquitin (Ub) or a Ub chain is attached to a substrate protein, is involved in diverse cellular processes in eukaryotes, including cell division, cell growth, signaling, apoptosis, and DNA repair (Pickart, 2001). Ubiquitination typically consists of three steps in forming an isopeptide bond, requiring the activities of three enzymes, named E1, E2, and E3 (Hershko and Ciechanover, 1998).

Substrate proteins can be ubiquitinated in different ways. In monoubiquitination, only one Ub molecule is attached to a target protein. Monoubiquitination has functions in histone regulation, endocytosis, and virus budding (Hicke, 2001). In polyubiquitination, substrate proteins are modified by various polyubiquitin chains (Haglund and Dikic, 2005; Pickart, 2001). Since Ub contains seven lysine (Lys, K) residues, K6, K11, K27, K29, K33, K48, and K63, a polyubiquitin chain can be linked through the C-terminal Gly76 of one Ub and one of the seven K residues of the next Ub. In Arabidopsis, diverse forms of polyubiquitin chains have been found (except for the K27-linked chain), with the K48-linked Ub chain being the most abundant, followed by K63- and K11-linked chains (Kim *et al.*, 2013; Maor *et al.*, 2007). It is well known that K48-linked polyubiquitination targets proteins for degradation by the 26S proteasome in both animals and plants (Vierstra, 2009). Some initial understanding has been gained regarding the E2s responsible for K63-linked ubiquitination and the role of K63-linked ubiquitination in plants (see below). However, little is known about the other forms of polyubiquitination in plants.

The Arabidopsis genome is predicted to encode approximately 37 E2s, which are grouped into 14 subfamilies (Bachmair *et al.*, 2001; Kraft *et al.*, 2005). This large number of E2s may reflect functional diversity in terms of different ubiquitination reactions (transferring a single Ub or forming polyubiquitin chains of various types) and interactions with different E3s and other factors required for their activities. On the other hand, the fact that some subfamilies have multiple members also suggests functional redundancy among the members (Callis, 2014). This suggestion is supported by available, albeit limited, experimental evidence.

It is important to understand the specific functions of each of the E2s. Although *in vitro* studies have shown that most of them possess the classical E2 activity in forming the E2-Ub complex through a thioester linkage, or could catalyze the formation of a Ub chain (reviewed by Callis, 2014), very limited information is available on the specific ubiquitination reactions in which the E2s are involved.

Further, only a few of the E2s have been characterized in terms of their biological functions. Arabidopsis UBC1 and UBC2, belonging to subfamily III, are homologs of the yeast RAD6, which is known to play a critical role in DNA repair and histone monoubiquitination for transcriptional activation (Hoege *et al.*, 2002; Kao *et al.*, 2004). UBC1 and UBC2, together with two closely related RING-type E3s called HUB1 (HISTONE MONOUBIQUITINATION1) and HUB2, have been shown to be involved in histone 2B monoubiquitination and regulation of flowering time (Cao *et al.*, 2008; Xu *et al.*, 2009). However, UBC3, the other member of subfamily III, does not show redundancy with UBC1 and UBC2, since only the *ubc1 ubc2* double mutant, and not double mutants with *ubc3*, showed the early flowering phenotype (Cao *et al.*, 2008).

UBC21 was initially identified as PEX4 through screening for mutants defective in peroxisomal processes (Zolman *et al.*, 2005). It is tethered to the peroxisome membrane through a peroxisomal protein, PEX22, and is suggested to function in the ubiquitination of the peroxisome matrix protein receptor PEX5. In yeast, Pex5 can be monoubiquitinated for translocation and polyubiquitinated, mainly through the K48-linked Ub chain, for degradation by a complex consisting of three E3 ligases, Pex2, Pex10, and Pex12 (Platta *et al.*, 2004). Studies of the Arabidopsis homologs of the E3 ligases indicate the existence of a similar mechanism in plants (Hu *et al.*, 2012). UBC24, one of the four members of subfamily IX, functions in inorganic phosphate (Pi) signaling and was originally identified as PHO2. *pho2*/*ubc24* mutant plants overaccumulate Pi in leaves and when grown in Pi-rich soil display signs of Pi toxicity such as chlorosis and necrosis (Aung *et al.*, 2006; Bari *et al.*, 2006). More recent studies have shown that UBC24/PHO2 works with an E3 named NLA (Nitrogen Limitation Adaptation) in polyubiquitination, with a Pi transporter, PT2, as a downstream target, leading to its degradation via the 26S proteasome (Park *et al.*, 2014). UBC32, a member of E2 subfamily XIV, was found to be a component of the endoplasmic reticulum-associated protein degradation (ERAD) complex, and to be induced by salt stress and involved in brassinosteroid-mediated growth promotion (Cui *et al.*, 2012). UBC32 has been shown to be involved in the polyubiquitination and degradation of a known ERAD substrate, MLO-12 (Cui *et al.*, 2012).

UBC35 and UBC36, two members of subfamily XV, are closely related to Ubc13 proteins of non-plant species, and they were able to complement the yeast *ubc13* mutant for spontaneous mutagenesis and sensitivity to DNA-damaging agents (Wen *et al.*, 2006); accordingly, these two E2s are also referred to as UBC13A and UBC13B, respectively. Ubc13 and its homologs are the only known E2s responsible for K63-linked ubiquitination in yeast and humans (Andersen *et al.*, 2008; Chen and Sun, 2009). Arabidopsis UBC13A (UBC35) has been shown to catalyze K63-linked ubiquitination *in vitro* (Wen *et al.*, 2006). The double mutant of Arabidopsis *UBC13A/B* displays strong phenotypes including shortened primary roots, a reduced number of lateral roots, and few and short root hairs (Li and Schmidt, 2010; Wen *et al.*, 2014). A tomato UBC13 homolog, Fni3, has been found to affect plant immunity positively (Mural *et al.*, 2013). Further, in yeast and humans, Ubc13 proteins require partnering with the ubiquitin-conjugating enzyme variant (UEV), which is a UBC domain-containing protein but lacks the catalytic cysteine. Four Arabidopsis UEV genes have been identified, with *UEV1D* implicated in the DNA damage response (Wen *et al.*, 2008). Results from these studies indicate that UBC13A/B (UBC35/36) have a function in different processes and likely catalyze K63-linked ubiquitination in plants, although *in vivo* evidence for involvement of Arabidopsis UBC13 protein in K63-linked ubiquitination remains to be obtained.

Apart from the E2s described above, little is known about the other E2s in plants, and there is a great need to understand them and their functions. To this end, in this study we characterized Arabidopsis UBC22, identified its important role in female gametophyte development, and obtained experimental evidence of its ability to catalyze a K11-specific ubiquitination reaction.

## Materials and methods

### Plant growth

*Arabidopsis thaliana* ecotype Columbia and its mutant lines were grown in a growth room or chamber (20 °C constant temperature, 16h day/8h night photoperiod with a fluence rate of 90±10 µmoles m^–2^ min^–1^). Arabidopsis *UBC22* T-DNA insertion lines SALK_011800 and GK_642C08 were obtained from the Arabidopsis Biological Resource Center (http://abrc.osu.edu) and Nottingham Arabidopsis Stock Centre (http://arabidopsis.info), respectively.

### Isolation and analysis of plant genomic DNA and RNA

Genomic DNA was isolated from Arabidopsis leaf tissues as described by Edwards *et al.* (1991) and 1.0 µl of the DNA sample was used in a standard 20 µl PCR, with the following program: 94 °C for 3min followed by 30 cycles of 94 °C for 45s, 55 °C for 45s, and 72 °C for 2min. The primers used are listed in Supplementary Table S2 at *JXB* online.

Total RNA was isolated using TRIzol reagent (Invitrogen) and RNA concentration was determined with a NanoVue Plus Spectrophotometer (GE Healthcare) following the manufacturer’s instructions. For reverse transcription (RT)-PCR, the first-strand cDNA was synthesized using the ThermoScript RT-PCR system (Invitrogen). The final input amount of cDNA used in the RT-PCR was normalized based on the level of the reference gene *At4g33380* (Czechowski *et al.*, 2005).

### Complementation of *ubc22* mutants

The full-length coding sequence of *UBC22* was amplified from wild-type (WT) Arabidopsis cDNA by RT-PCR with the primers HW1089 and HW1090. The fragment was cloned into a plant expression vector modified from *pCambia1300* (http://www.cambia.org/daisy/cambia/585.html) and carrying an HA (influenza hemagglutinin) protein fusion tag, resulting in the construct *Pro35S::HA-UBC22*. A 3.2kb promoter region (–3219 to –18 positions upstream of ATG) of *UBC22* was amplified from WT Arabidopsis genomic DNA by PCR and cloned as a HindIII-BamHI fragment to replace the 35S promoter in *Pro35S::HA-UBC22*, resulting in *ProUBC22::HA-UBC22*. The construct was then introduced into *ubc22-1* mutant plants, and progeny (T2) plants were used in the phenotype analyses.

### Light microscopy

For observation of female gametophytes, the pistil was removed and cuts made on both sides of the pistil replum using a scalpel to expose the ovules. The samples were fixed in FAA solution (1:2:10:7 formaldehyde:acetic acid:ethanol:water) for 1h, washed in 10–20% ethanol three times, each for 5–10min, and transferred into water. The ovules were detached, mounted in clearing solution (8:2:1 chloral hydrate:water:glycerol) and observed under a Leica DM2500 microscope equipped with differential interference contrast (DIC) optics. Photographs were taken using a Leica DFC450C digital microscope camera.

### Histochemical analysis

For preparing a GUS (β-glucuronidase) fusion construct, first, a HindIII-EcoRI fragment containing a promoter::GUS reporter was cut from a pBI121-based construct and cloned into *pCambia1300*. The 3.2kb *UBC22* promoter region was cloned into a HindIII-BamHI fragment to replace the existing promoter in the modified *pCambia1300*, resulting *ProUBC22::GUS*. This construct was introduced into WT plants. Transformants were selected on half-strength Murashige and Skoog plates containing 1% sucrose, 0.7% phytoagar, and 40 μg/ml hygromycin. The plates were placed in a tissue culture chamber. A number of transformants were transferred to, and grown in, soil. Histochemical GUS staining was performed as described by Jefferson (1987) with minor modifications. Seedlings and flowers were vacuum-infiltrated for 1min in GUS staining solution [50mM PO_4_^3−^ (pH 7.0), 2mM K_3_(Fe(CN)_6_), 2mM K_4_(Fe(CN)_6_), 10mM Na_2_EDTA (pH 8.0), 0.08% Triton X-100, 0.5mg/ml X-gluc] and incubated overnight at 37 °C. The pistils were first fixed in 3.7% formaldehyde in GUS staining solution for 15min and then washed with GUS staining solution for 5–10min. They were then incubated at 37 °C for 12–16h (ovules at early stages of development for approximately 12h and ovules at late stages of development, e.g. FG7, for 16h). After fixation in FAA solution for 10min and washing with 10–25% ethanol three times, each for 5–10min, ovules were mounted in the clearing solution (described above) and examined under the microscope equipped with DIC optics.

### Protein expression and purification

The coding sequence of *UBC22* was cloned into *pET28c* (Novagene). The resulting construct *pET28-UBC22* was used to transform *Escherichia coli* strain BL21 (DE3) (Stratagene). For protein expression, 2.5ml of overnight culture was added to 100ml fresh Luria-Bertani liquid medium containing 50 μg/ml kanamycin. The culture was grown at 37 °C with vigorous shaking until the optical density at 600nm reached approximately 0.6; then, isopropyl β-D-1-thiogalactopyranoside (IPTG) was added to a final concentration of 0.5mM, and the culture was grown for another 4h. Cells were then spun down and resuspended in lysis buffer [50mM PO_4_^3−^ (pH 8.0), 300mM NaCl, 10mM imidazole]. Lysozyme (Fisher, BP535-1) was added to a final concentration of 1mg ml^–1^ and nuclease (Piece Universal Nuclease through Fisher Scientific) was added to a concentration of 3 units ml^–1^ culture, and the suspension was incubated on ice for 15–30min. The lysate was centrifuged at 12000 × *g* for 20min at 4 °C and the recombinant protein was purified from the supernatant using Ni-NTK spin columns (Qiagen) following the manufacturer’s instructions.

### Ubiquitin conjugation reaction

*In vitro* Ub conjugation reactions were performed using the purified His-tagged UBC22 as described above. The Ub thioester/conjugation initiation reagents were purchased from Boston Biochem (K-995). Unless noted otherwise, the reaction mixture contained 5 μM purified His-UBC22 and 62.5 μM Ub in the supplied reaction buffer; concentrations of other components were used following the manufacturer’s instructions. K11R, K48R, and K63R mutant Ub proteins were purchased from Boston Biochem (UM-K11R, UM-K48R, and UM-K63R). The conjugation reactions were performed at 30 °C for 4h. Samples were added with 4X non-reducing loading buffer [200mM Tris-HCl (pH 6.8), 8% SDS, 0.2% bromophenol blue, 40% glycerol] and heated at 95 °C for 5min, then subjected to SDS-PAGE (15% gel). Ub and Ub dimers were detected by western blotting using a monoclonal mouse anti-Ub antibody (P4D1, Cell Signaling Technology) and a horseradish peroxidase-conjugated goat anti-mouse antibody (Bio-Rad). The signal was visualized with ECL Prime reagent (GE Health) according to the manufacturer’s instructions.

## Results

### Characterization of Arabidopsis UBC22 and *ubc22* mutants

The 37 Arabidopsis E2s are grouped into 14 subfamilies, many of which have more than one member (Kraft *et al.*, 2005). UBC22 (At5g05080) is the sole member of subfamily X. Interestingly, sequence analysis showed that Arabidopsis UBC22 is more closely related to a human E2, Ube2S, than to other Arabidopsis E2s (Supplementary Fig. S1), and it shares 65% sequence identity with the human and animal Ube2S homologs (Supplementary Fig. S2).

**Fig. 1.**
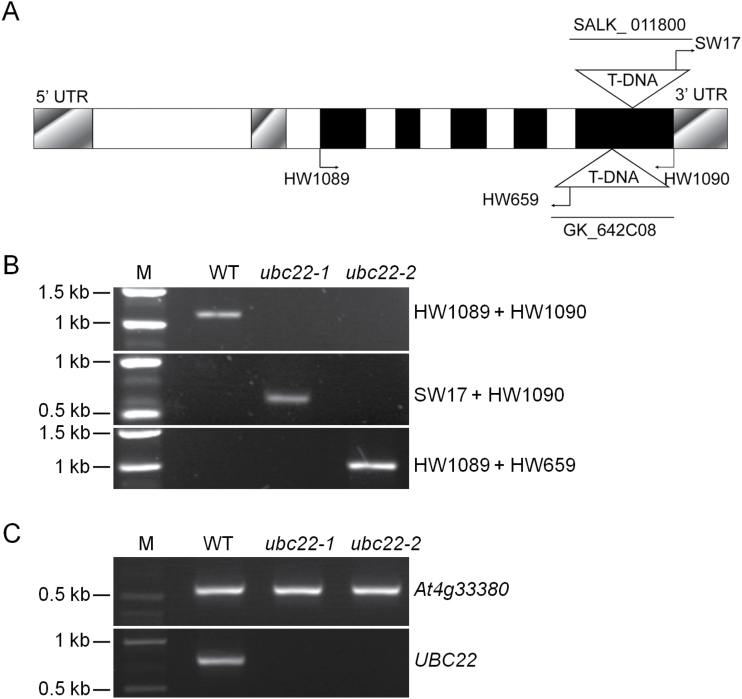
**Conﬁrmation of *ubc22* T-DNA insertion mutants.** (A) Schematic representation of the genomic structure of *UBC22* with the locations of primers and insertion sites of T-DNA in the *ubc22-1* and *ubc22-2* mutants shown. Closed boxes, exons; open boxes, introns; shadowed boxes, untranslated regions; HW1089 and HW1090, gene-speciﬁc primers for *UBC22*; SW17, T-DNA left border primer for SALK_011800 line; HW659, T-DNA left border primer for GK_642C08 line. (B) Characterization of *ubc22* T-DNA lines by genomic PCR. Plant lines used are indicated above the panels, and the primers to the right of the panels. M, DNA size marker. (C) Characterization of *ubc22* T-DNA lines by RT-PCR; 30 PCR cycles were used. PCR amplification of a reference *At4g33380* cDNA is shown in the upper panel and amplification of *UBC22* cDNA is shown in the lower panel.

To investigate the function of Arabidopsis *UBC22*, a T-DNA insertion line (SALK_011800) was obtained from the Arabidopsis Biological Resource Center and genotyped to obtain homozygous lines. Since phenotypes were observed with this line (see below), another independent T-DNA line (GK_642C08) was obtained from the Nottingham Arabidopsis Stock Centre. Sequence analysis of these two T-DNA insertion lines revealed that the T-DNA was in the fifth exon of *UBC22* in both lines (Fig. 1A). Genomic DNA PCR and RT-PCR using *UBC22* gene-specific primers did not amplify the full-length genomic or cDNA of *UBC22* (Fig. 1B, C). Thus, the two alleles are considered null mutation alleles, and are designated *ubc22-1* (SALK_011800) and *ubc22-2* (GK_642C08) in this study.

**Fig. 2.**
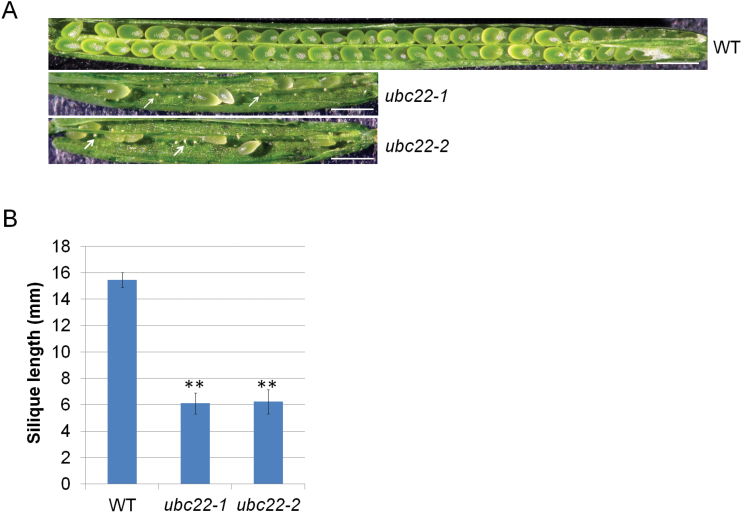
**Silique phenotype of *ubc22* mutants.** (A) Representative siliques of WT, *ubc22-1*, and *ubc22-2* mutant plants. The white arrows indicate aborted ovules. Scale bar, 1mm. (B) Silique length of WT, *ubc22-1*, and *ubc22-2* mutant plants. Eight fully elongated siliques of the main inflorescent stem from each plant and four different plants in each line were measured. Student’s *t*-test was performed to determine the differences between the mutants and WT. ** Significant difference at *P*<0.01.

To characterize the phenotypes of *ubc22-1* and *ubc22-2*, plants from these two T-DNA lines were grown side by side with WT plants. While the mutant plants were similar to the WT plants in most aspects, they had considerably shorter siliques than the WT plants (less than 50% of WT length) (Fig. 2A, B). Further examination showed that approximately 88.3% of ovules were aborted in the mutant plants (Fig. 2A, Table 1), whereas the total ovule number was not affected (Table 1). Reciprocal crosses between the WT and mutant revealed that the short silique phenotype was retained when the mutant, but not the WT, was used as the female parent (Table 2).

**Table 1.** Analysis of ovule and seed development in WT and ubc22-1 mutant plants

Line	Seeds/ovules counted	Normal seeds	Aborted ovules	Aborted seeds	Number of ovules per silique	Number of seeds per silique
WT	1536 (100%)	1531 (99.67%)	4 (0.26%)	1 (0.07%)	61.4±2.6	61.2±2.7
*ubc22-1*	1515 (100%)	156 (10.30%)	1337 (88.25%)	22 (1.45%)	60.6±3.1	6.2±2.1

Five WT and mutant plants were used, and for each plant the first five siliques were analyzed. For the number of ovules and number of seeds per silique, the data shown are mean±SD. The difference between the WT and the mutant in number of seeds per silique was significant (*P*<0.01).

**Table 2.** Analysis of seed development in siliques from reciprocal crosses and self-fertilized siliques of heterozygous plants

Female × male	Seeds/ovules counted	Normal seeds	Aborted ovules	Aborted ovules per silique	Aborted seeds	Silique length (mm)
WT × WT	1265 (100%)	1264 (99.92%)	1 (0.08%)	0.1±0.2	0	16.2±0.2
*ubc22-1* × WT	1165 (100%)	89 (7.64%)	1057 (90.73%)	52.9±2.9**	19 (1.63%)	6.4±1.0
WT × *ubc22-1*	1228 (100%)	1154 (93.97%)	71 (5.78%)	3.6±2.5**	3 (0.24%)	15.7±0.6
*ubc22-1/+* × *ubc22-1/+* (self-fertilized)	1196 (100%)	1038 (86.79%)	157 (13.13%)	7.9±2.9**	1 (0.08%)	14.9±0.6

Reciprocal crosses were made between WT and homozygous *ubc22-1* mutant plants. Heterozygous plants (*ubc22-1/+*) were also included in the analysis as a comparison. Twenty fully elongated siliques from each type were used for determining the number of normal seeds, aborted ovules, and aborted seeds, as well as silique length. Student’s *t*-test was performed on the number of aborted ovules per silique from reciprocal crosses and heterozygous plants compared with WT plants. ** Significant difference at *P*<0.01.

We analyzed the seed setting. The reciprocal cross using the *ubc22-1* mutant as the female parent had only approximately 7.6% of ovules developing into normal seeds, while the reciprocal cross using WT as the female parent showed approximately 94% of ovules developing into normal seeds, in comparison to almost 100% in the WT plants. These results confirmed that the major defect was due to the female parent. Further, in the self-pollinated siliques of heterozygous plants, approximately 13.1% of ovules were aborted (Table 2); this proportion was much less than half of that in the self-pollinated siliques of the mutant (88.3%; Table 1), suggesting that majority of female gametophytes carrying the mutant allele could develop normally in the heterozygous plants. In addition, when the WT was used as the female parent and the mutant as the male parent, the siliques contained only 5.8% aborted ovules (Table 2). Thus, we analyzed the mutant pollen and observed that the homozygous mutant plants had a slightly higher frequency of pollen with only one or no sperm nuclei (2.8% *versus* 1.6% in the WT) (Supplementary Fig. S3 and Table S1). These results indicate a minor defect in male gametogenesis of the mutant.

**Fig. 3.**
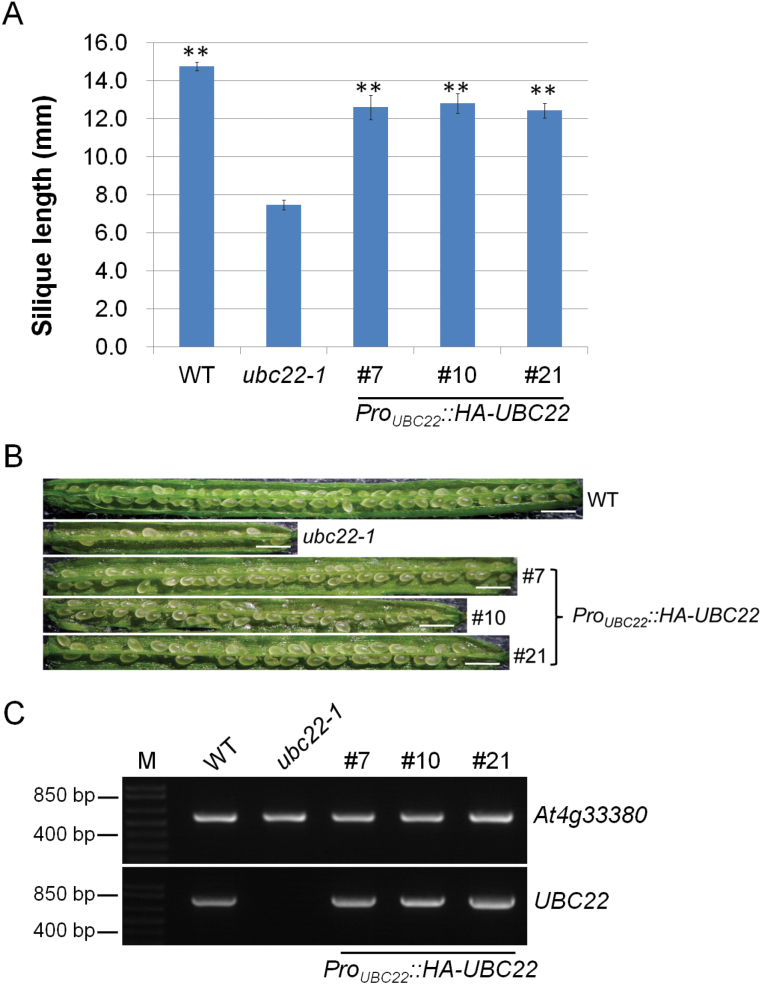
**Complementation of the *ubc22-1* mutant by *Pro***_***UBC22***_ ***::HA-UBC22*.** For complementation, *Pro*_*UBC22*_ *::HA-UBC22* was introduced into the *ubc22-1* mutant. T2 plants of independent lines were used for further analysis. (A) Silique length of WT, *ubc22-1*, and three individual complementation lines (#7, #10 and #21). Approximately six siliques from each plant were measured, from at least four different plants in each line. Student’s *t*-test was performed to determine whether there was a significant difference in silique length between the complementation lines and the mutant. ** Significant difference at *P*<0.01. (B) Representative siliques from WT, *ubc22-1*, and three complementation lines. Scale bar, 1mm. (C) RT-PCR analysis of WT, *ubc22-1*, and three independent complementation lines. PCR amplification of a reference *At4g33380* cDNA is shown in the upper panel and amplification of *UBC22* cDNA is shown in the lower panel. Plant lines used are indicated above the panels. M, DNA size marker.

The observation that two independent T-DNA insertion lines showed nearly identical phenotypes strongly suggests that the phenotypes were due to the inactivation of *UBC22*. To confirm that the phenotype was due to inactivation of *UBC22*, we expressed *UBC22* driven by its own promoter in the *ubc22-1* mutant to see whether it could complement the mutant phenotypes. Among the 21 T1 transformants obtained, three independent transformants showed nearly full complementation. Quantitative measurements using T2 plants of these lines showed that the silique length in those lines was approximately 85% of that in the WT plants (Fig. 3A). In addition, the majority of the seeds in these HA-UBC22 lines developed normally (Fig. 3B). The transcript level of *HA-UBC22* in these lines was found to be similar to the level of endogenous *UBC22* in the WT plants (Fig. 3C).

### Female gametophyte development in the *ubc22* mutant

**Fig. 4.**
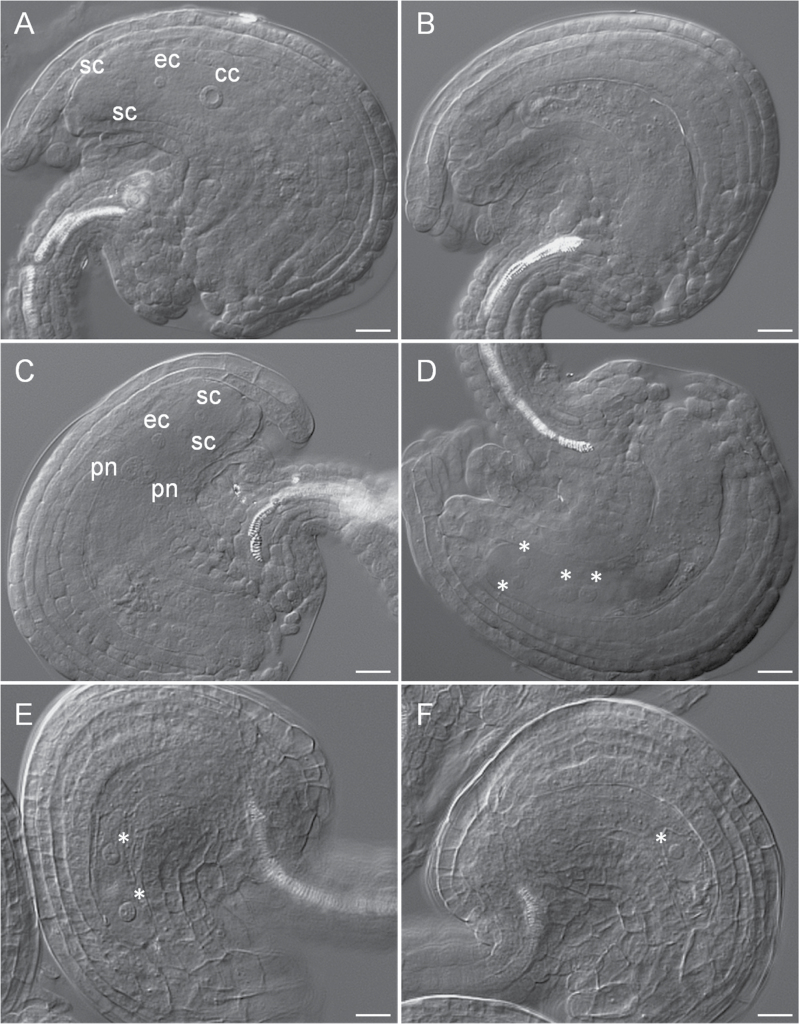
**Female gametophyte development in WT and *ubc22-1*.** Ovules from flowers just before opening were prepared and observed under a microscope with DIC. (A) A typical WT embryo sac (FG7 stage) showing one central cell (cc), one egg cell (ec), and two synergid cells (sc). (B–F) Abnormal embryo sacs in *ubc22-1* plants. (B) Mutant embryo sac without any nucleus. The embryo sac was smaller and narrower than the WT embryo sac. (C) Mutant embryo sac at the FG5 stage showing two polar nuclei (pn), two synergid cells, and one egg cell. (D) Mutant embryo sac at the FG4 stage, with four nuclei (indicated by asterisks). (E) Mutant embryo sac at the FG3 stage, with two nuclei and a larger vacuole between them. (F) Mutant embryo sac with only one nucleus. Scale bars, 10 μm.

To further characterize the ovule abortion phenotype of the mutant, the development of the female gametophyte in *ubc22-1* mutant plants was examined. First, embryo sacs just before flowering were investigated. In the WT Arabidopsis plants, the typical embryo sac at FG7, with egg, central, and synergid cells (Christensen *et al.*, 1997), could be easily distinguished under a microscope with DIC (Fig. 4A). By contrast, in the majority of mutant embryo sacs (63.8%; Table 3), no egg, central, and synergid nuclei could be seen. Generally, these embryo sacs were smaller and narrower than the WT embryo sacs. In addition, the contents of the mutant embryo sacs appeared grainier under the microscope (Fig. 4B). These cellular features suggest that either there was no gametogenesis or the gamete nuclei had degenerated in these embryo sacs. Most of the mutant embryo sacs with observable gamete nuclei were also abnormal. In terms of developmental stage, only a small proportion of them (5.7%) were at the typical FG7 stage, with one central, one egg, and two synergid nuclei, similar to the WT embryo sacs (Fig. 4A). A slightly larger proportion (7.0%) were at the FG5 stage, with two polar, one egg, and two synergid nuclei (Fig. 4C). Some embryo sacs were still at the FG4 stage, with four nuclei and one large vacuole (Fig. 4D).

**Table 3.** Female gametophyte development in WT and ubc22-1 mutant plants

Line	Total number	Female gametophyte stage							No nucleus	One nucleus	Two nuclei
		FG1	FG2	FG3	FG4	FG5	FG6	FG7			
WT (%)	270 (100)	0	0	0	0	1 (0.4)	0	268 (99.3)	1 (0.4)	0	0
*ubc22-1* (%)	331 (100)	2 (0.6)	2 (0.6)	4 (1.2)	8 (2.4)	23 (7.0)	3 (0.9)	19 (5.7)	211 (63.8)	47 (14.2)	12 (3.6)

Embryo sacs from flowers just before opening were dissected, prepared, and then observed under a microscope equipped with DIC optics. The stages of embryo sacs were determined based on the number of cells and their configurations according to the description of Christensen *et al.* (1997).

**Fig. 5.**
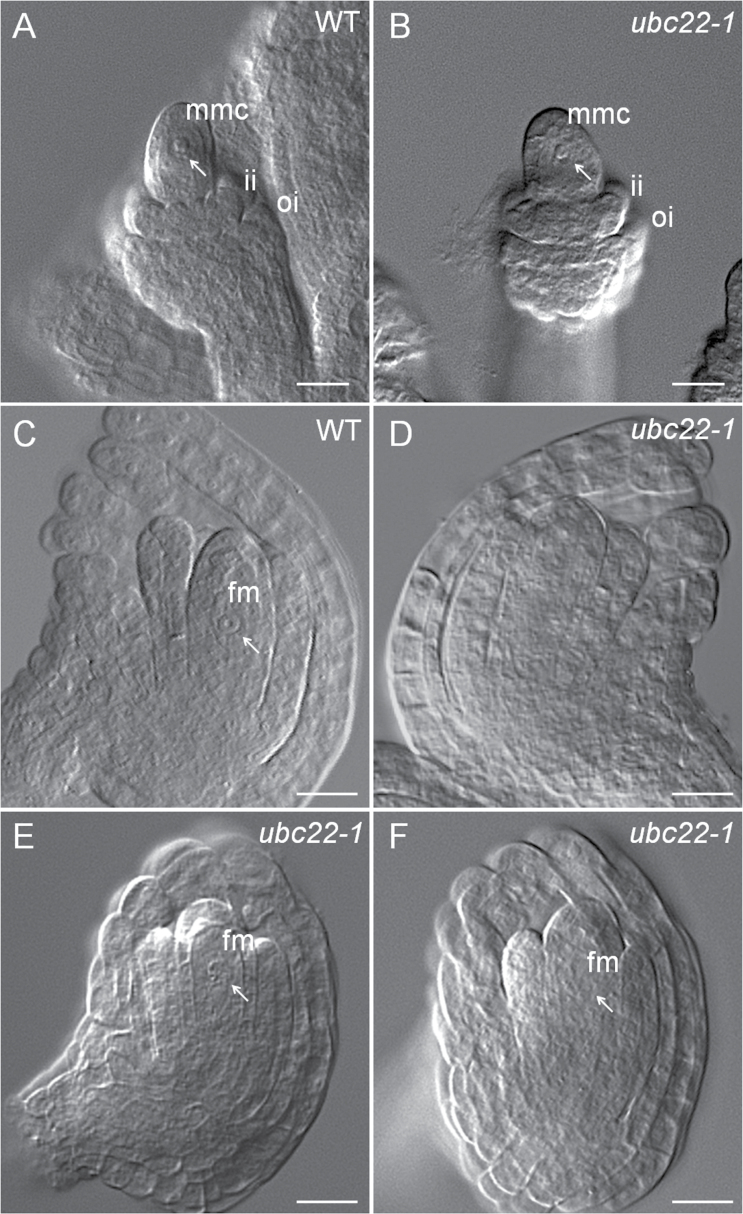
**Megaspore mother cell (MMC) and functional megaspore (FM) development in WT and *ubc22-1*.** Ovules from the flower buds were prepared and observed under a microscope with DIC. (A, B) WT (A) and *ubc22-1* mutant (B) ovules with a typical MMC. ii, inner integument; oi, outer integument. (C–F) Ovules at the FM stage. (C) WT ovule with a typical FM. (D) A *ubc22-1* ovule without an observable FM. (E, F) *ubc22-1* ovules with an abnormal FM. The FM in (E) is visible, but the boundary is not clear and the shape is abnormal. The FM in (F) is barely visible under the microscope. White arrows indicate the MMC in (A, B) or the FM in (C, E, F). Scale bars, 10 μm.

The ovules at earlier stages of development were examined to determine the stage at which ovule development of the mutants departed from that of WT. During megasporogenesis, a megaspore mother cell (MMC) is produced through differentiation of one subepidermal archesporial cell. Careful examination of MMC development in ovules at stage 2-II (Schneitz *et al.*, 1995), with the inner integument initiating, did not reveal any obvious difference between WT and mutant ovules (Fig. 5A, B, Table 4). Therefore, the development of the functional megaspore (FM) was examined. In the WT ovule, following meiosis, the megaspore closest to the chalazal end enlarges and gives rise to the FM, whereas the other three megaspores degenerate. We examined ovules at about stage 3-I according to Schneitz *et al.* (1995), with the inner integument near and just below the top of the nucellus. Most of the WT ovules examined contained a typical FM, with the nucleus having a clear edge under DIC, a round shape, and often an easily recognizable nucleolus (Fig. 5C). In contrast, in approximately 37% of the mutant ovules examined, no FM nucleus was observed (Table 5, Fig. 5D). In 35% of the mutant ovules, FMs could be identified but appeared abnormal; their nuclei did not have an easily recognized boundary, appeared thin in nuclear content, and/or were not spherical in shape (Fig. 5E). In some cases, the nuclei of FMs appeared thin and ‘flat’ under DIC, with the nuclear–cytoplasm boundary barely visible (Fig. 5F), suggesting that they might be at late stages of degeneration. These data indicate that the FM in some mutant ovules was undergoing degeneration or had degenerated, resulting in no nucleus being observed. Thus, these results indicate that the abnormality during female gametophyte development in the *ubc22-1* mutant likely started with the degeneration of the FM in some ovules.

**Table 4.** Megaspore mother cell (MMC) development in WT and ubc22-1 mutant ovules

Line	Total number	No nucleus	One nucleus	Two nuclei
WT	267 (100%)	40 (15.0%)	211 (79.0%)	16 (6.0%)
*ubc22-1*	138 (100%)	20 (14.5%)	112 (81.2%)	6 (4.3%)

Flowering buds were first checked for ovule developmental stage, and the sixth bud from the newly opened flower was used. The buds were dissected, prepared, and then observed under a microscope equipped with DIC optics. The number of ovules in different categories, in terms of the presence and appearance of MMC, was determined.

**Table 5.** Functional megaspore (FM) development in WT and ubc22-1 mutants

Line	Total number	Typical FM (Type I)	Visible FM with abnormalities (Type II)	FM not observable (Type III)
WT (%)	114 (100)	99 (86.8)	10 (8.8)	5 (4.4)
*ubc22-1* (%)	123 (100)	34 (27.6)	43 (35.0)	46 (37.5)

Flowering buds (the fifth bud from the newly opened flower) were dissected, prepared, and then observed under a microscope equipped with DIC optics. Ovules at the functional megaspore (FM) stage (with the inner integument near, but below, the top of the nucellus) were included in the analysis. Three types of ovules were observed. Type I (normal): A typical FM had one prominent nucleus with a round shape and clear nuclear–cytoplasm boundary. Type II: The FM was visible. However, the nuclear–cytoplasm boundary was not clear, and/or the nucleoplasm appeared to be disorganized. In the *ubc22-1* mutant, the FM was often barely visible (see Fig. 5E, F). Type III: No FM could be observed.

### Expression of *UBC22* in different tissues and developmental stages

To understand the expression of *UBC22*, we first examined the microarray data available from Genevestigator (Zimmermann *et al.*, 2004). As shown in Supplementary Fig. S4, *UBC22* is expressed in different tissues, with a higher level of expression in seeds. To determine the locations of *UBC22* expression more specifically, we fused its promoter to the GUS reporter and the patterns of expression were analyzed by histochemical GUS staining. In 10-day-old seedlings, a strong GUS signal was detected in vascular tissues, root tips, and lateral root primordia (Supplementary Fig. S5A–C). Similar results were obtained from 20-day-old seedlings. In the reproductive organs, a GUS signal was observed in stigmas, filaments, and veins of sepals, but very little was detected in petals and pollen (Supplementary Fig. S5D). Additionally, a GUS signal was detected in ovules of different developmental stages (Supplementary Fig. S5E–G). These data indicate that *UBC22* is expressed in a variety of tissues.

### UBC22 catalyzing K11-linked ubiquitin dimer formation *in vitro*

**Fig. 6.**
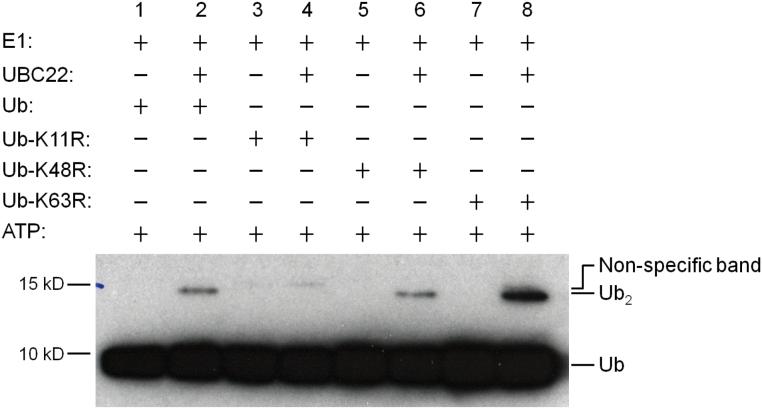
**Ub dimer formation assay by His-UBC22.** An *in vitro* Ub dimer formation assay was performed with (lanes 2, 4, 6, and 8) or without (lanes 1, 3, 5, and 7) the addition of His-UBC22. The components added in the different reactions are indicated at the top of the figure. The conjugation reactions were performed at 30 °C for 4 hours. Assay samples were subjected to SDS-PAGE. Ub and Ub dimer were detected by western blotting using an anti-Ub antibody. Free Ub and Ub dimer (Ub_2_) are indicated at the right side of the figure. For the K11R mutant, no new Ub dimer formation was seen when His-UBC22 protein was added. Due to slight impurity in the recombinant K11R protein, one weak band was present which is slightly higher than the Ub dimer synthesized when His-UBC22 was added. Three experiments were performed and produced similar results.

The observation that Arabidopsis UBC22 is more closely related to human Ube2S (65% identity) than to other Arabidopsis E2s (Supplementary Fig. S1) suggests functional conservation between the Arabidopsis and human proteins. In addition, the N-terminal region of approximately 160 amino acids shares high levels of similarity with the human Ube2S and putative Ube2S homologs from other species (Supplementary Fig. S2). It was recently shown that human Ube2S is capable of generating K11-linked Ub dimers and chains (Wickliffe *et al.*, 2011a). We thus determined whether UBC22 could catalyze Ub dimer formation *in vitro* using recombinant UBC22. As shown in Fig. 6 (lane 2), Ub dimers were formed when His-UBC22 was present in the reaction. Further, when the Ub-K11R mutant, which lacks the K11 residue, was used (lanes 3 and 4 in Fig. 6), little dimer was produced; in contrast, dimer formation was not affected when the Ub-K63R mutant, which lacks the K63 residue, was used (lanes 5 and 6 in Fig. 6). These results indicate that UBC22 was able to catalyze dimer formation *in vitro* specifically through the K11 residue of Ub.

## Discussion

### *UBC22* plays an important role in female gametophyte development

During megasporogenesis, the MMC develops from one subepidermal archesporial cell. In the great majority of angiosperms, following meiosis, the megaspore closest to the chalazal end enlarges and gives rise to the FM, while the other three megaspores degenerate (Yadegari and Drews, 2004). The developmental feature that only one megaspore survives ensures that only one egg cell and one central cell will be produced in each ovule. In the *ubc22* mutants in this study, the MMC developed normally, based on the observations of developing ovules at stage 2_II (Schneitz *et al.*, 1995); however, approximately 37% of ovules at stage 3-I (with the outer integuments near the top of nucellus) did not have an FM. In a small proportion of WT ovules (4.4%), the FM could not be observed; this was likely due to developmental variation among ovules or simply a limitation of the microscope optics. Since an FM was present in most of the WT ovules at this developmental stage, but absent in a large proportion of the mutant ovules, we infer that a key point for UBC22 function is during female megasporogenesis, specifically from MMC to the development of the FM.

There are two possible causes for the absence of FMs in the *ubc22* mutant: (i) arrest or inhibition of meiosis and (ii) degeneration of the FM together with the other three megaspores. Inhibition of meiosis usually leads to abnormal meiosis and meiotic products, which were not observed in the *ubc22* mutant. It has been shown that the non-functional megaspores degenerate, likely through programmed cell death (Citterio *et al.*, 2005). In the *ubc22* mutant, many FMs appeared to be at different stages of degeneration, from having an indistinct nuclear–cytoplasm boundary to almost completely disappearing (Fig. 5E, F). This observation, together with the absence of an FM in a large percentage of ovules, suggests that the FM degenerates along with the three non-functional megaspores in the mutant.

In addition to the absence of an FM, defects also occurred during female gametogenesis in the *ubc22* mutant. Over 60% of mutant embryo sacs at the flowering stage did not contain any gamete nuclei, likely due to degeneration during either megasporogenesis or gametogenesis. Further, in approximately 30% of embryo sacs in which gamete nuclei were observed, the embryo sacs were at various stages of gametogenesis, with a relatively high proportion in stage FG5 (Table 3), in contrast to the WT embryo sacs, which were almost all at stage FG7; this result indicates that megagametogenesis in those mutant ovules was delayed to various extents. The defects in female megasporogenesis and gametogenesis in the *ubc22* mutant indicate that *UBC22* plays a critical role in female gametophyte development. It could be postulated that the inactivation of UBC22 inhibits female gametophyte development, likely due to impaired degradation of certain substrate proteins. Since the extent of inhibition varies among ovules, it is possible that strong inhibition in some ovules leads to degeneration at the FM stage, while weaker inhibition allows FM development but affects female gametogenesis.

Although major defects were observed in female gametophyte development in the *ubc22* mutant plants, the results of reciprocal crossing experiments and the analysis of mutant pollen also revealed a relatively minor effect of *UBC22* inactivation on male gametogenesis. In addition, the results from promoter-GUS expression (Fig. 6) and microarray data (Supplementary Fig. S4) showed that *UBC22* is expressed in various tissues, suggesting that UBC22 may have a function in other tissues. The widespread expression of *UBC22* also provides an explanation for the influence of heterozygous sporophytic tissues on the development of female gametophytes carrying the mutant gene.

### UBC22 likely catalyzes K11-linked protein ubiquitination

UBC22 is the sole member of subfamily X among the 37 Arabidopsis E2s (Kraft *et al.*, 2005), implying that it has a unique function in plants. Several studies have suggested that UBC22 may be involved in polyubiquitination in Arabidopsis and could catalyze E3-independent ubiquitination *in vitro* (Kraft *et al.*, 2005; Takahashi *et al.*, 2009; Zhao *et al.*, 2013). Additionally, an E2 Ub conjugation assay showed thioester linkage formation between Ub and UBC22 (Zhao *et al.*, 2013). The present results further show that UBC22 can catalyze Ub dimer formation *in vitro* independent of the presence of an E3.

The close sequence similarity of Arabidopsis UBC22 with the human and animal Ube2S homologs (Supplementary Fig. S1) suggests functional conservation. Results from different studies have shown that human Ube2S plays a unique function in K11-linked polyubiquitination together with the anaphase-promoting complex/cyclosome (APC/C), a large complex consisting of approximately 13 subunits, which targets cell cycle proteins for degradation by the 26S proteasome (Garnett *et al.*, 2009; Wickliffe *et al.*, 2011a; Williamson *et al.*, 2009). So far, Ube2S is the only E2 that has been shown to be able to catalyze K11-linked ubiquitination. A major role for K11-linked ubiquitination is in the regulation of mitosis (Wickliffe *et al.*, 2011b). In this study, we showed that the formation of Ub dimer *in vitro* is K11-specific. This finding, together with the sequence conservation across UBC22 and the mammalian Ube2S homologs, strongly supports the notion that UBC22 catalyzes K11-linked ubiquitination in plants.

APC/C acts as a platform to recruit substrates to E2s for their polyubiquitination. In mammalian cells, Ube2S conjugates the C-terminus of a donor Ub to the K11 of an acceptor Ub (Wickliffe *et al.*, 2011a). APC/C provides acceptor-Ub binding sites for Ube2S. The APC/C is highly conserved in plants, and different APC/C subunits from Arabidopsis are able to complement the corresponding yeast mutants (Capron *et al.*, 2003; Eloy *et al.*, 2011; Wang *et al.*, 2012). Results from studies in Arabidopsis indicate that APC/C functions in regulating the cell cycle by targeting cell cycle proteins containing the specific D- or KEN/GxEN-box destruction signals (Heyman and De Veylder, 2012). In Arabidopsis, the APC/C consists of at least 11 components, and some Arabidopsis APC/C mutants display defects in female gametogenesis. The ovules of *apc2* and *apc6* mutants are arrested at the two-nucleus stage of megagametogenesis (Capron *et al.*, 2003; Kwee and Sundaresan, 2003), while defects at different stages of megagametogenesis are found in *apc1* and *apc4* mutants (Wang *et al.*, 2012, 2013). Thus, it would be interesting to determine whether Arabidopsis UBC22 interacts with the APC/C. However, there are important differences between the phenotypes of *ubc22* mutants and *apc* mutants. *ubc22* mutants show clear defects in both megasporogenesis and megagametogenesis, while the reported defects in *apc* mutants are only in megagametogenesis. Thus, it is unlikely that the *ubc22* mutant phenotypes are entirely due to impaired ubiquitination through the APC/C.

In humans, Ube2S is considered to be an ‘elongation’ E2, which extends the Ub chain through K11-linkages following the initial ubiquitination of the substrate by another E2 (Ube2C/UbcH10 or Ube2D/UbcH5) together with APC/C (Primorac and Musacchio, 2013). In Arabidopsis, UBC19 and UBC20 have been identified as the homologs of UbcH10, based on sequence relatedness and their ability to complement a yeast mutant (Criqui *et al.*, 2002). However, currently there is no information regarding the specific ubiquitination processes with which UBC19 and UBC20 are involved in plants. The present results have provided initial experimental evidence for the existence of the atypical K11-linked form of protein ubiquitination in plants, and identified a critical role for UBC22 in female gametophyte development. It will be interesting to determine whether a module similar to the animal Ube2S-APC/C module is conserved in plants, as well as possible differences between the plant and animal E2 homologs involved in K11-specific ubiquitination.

## Supplementary data

Figure S1. Phylogenetic analysis of Arabidopsis E2s.

Figure S2. Alignment of putative UBC22 homologs from different species.

Figure S3. Pollen of WT and *ubc22* mutants.

Figure S4. Developmental expression profile of *UBC22*.

Figure S5. GUS staining of transgenic *Pro*_*UBC22*_ *::GUS* plants.

Table S1. Analysis of pollen development in WT and *ubc22-1* mutant plants.

Table S2. Sequences of primers used for confirming *ubc22* mutants and transcript level of *UBC22* in the complementation lines.

Supplementary Data
