# Supplementary material for: Arabidopsis ubiquitin-conjugating enzyme UBC22 is required for female gametophyte development and likely involved in Lys11-linked ubiquitination
Source: J Exp Bot. 2016 Apr 10;67(11):3277–88. doi: 10.1093/jxb/erw142 (PMC4892721; doi:10.1093/jxb/erw142)
Supplement: Supplementary Data [file supp_67_11_3277__index.html]

Arabidopsis ubiquitin-conjugating enzyme UBC22 is required for female gametophyte development and likely involved in Lys11-linked ubiquitination — Arabidopsis ubiquitin-conjugating enzyme UBC22 is required for female gametophyte development and likely involved in Lys11-linked ubiquitination — Supplementary Data 

# Arabidopsis ubiquitin-conjugating enzyme UBC22 is required for female gametophyte development and likely involved in Lys11-linked ubiquitination

## Supplementary Data

Data files

- supplementary\_figures\_S1\_S5\_tables\_S1\_S2.pdf - Supplementary Data
