# Supplementary material for: Arabidopsis ubiquitin-conjugating enzyme UBC22 is required for female gametophyte development and likely involved in Lys11-linked ubiquitination
Source: J Exp Bot. 2016 Apr 10;67(11):3277–88. doi: 10.1093/jxb/erw142 (PMC4892721; doi:10.1093/jxb/erw142)
Supplement: Supplementary Data [file supp_erw142_supplementary_figures_S1_S5_tables_S1_S2.pdf]

SUPPLEMENTARY INFORMATION

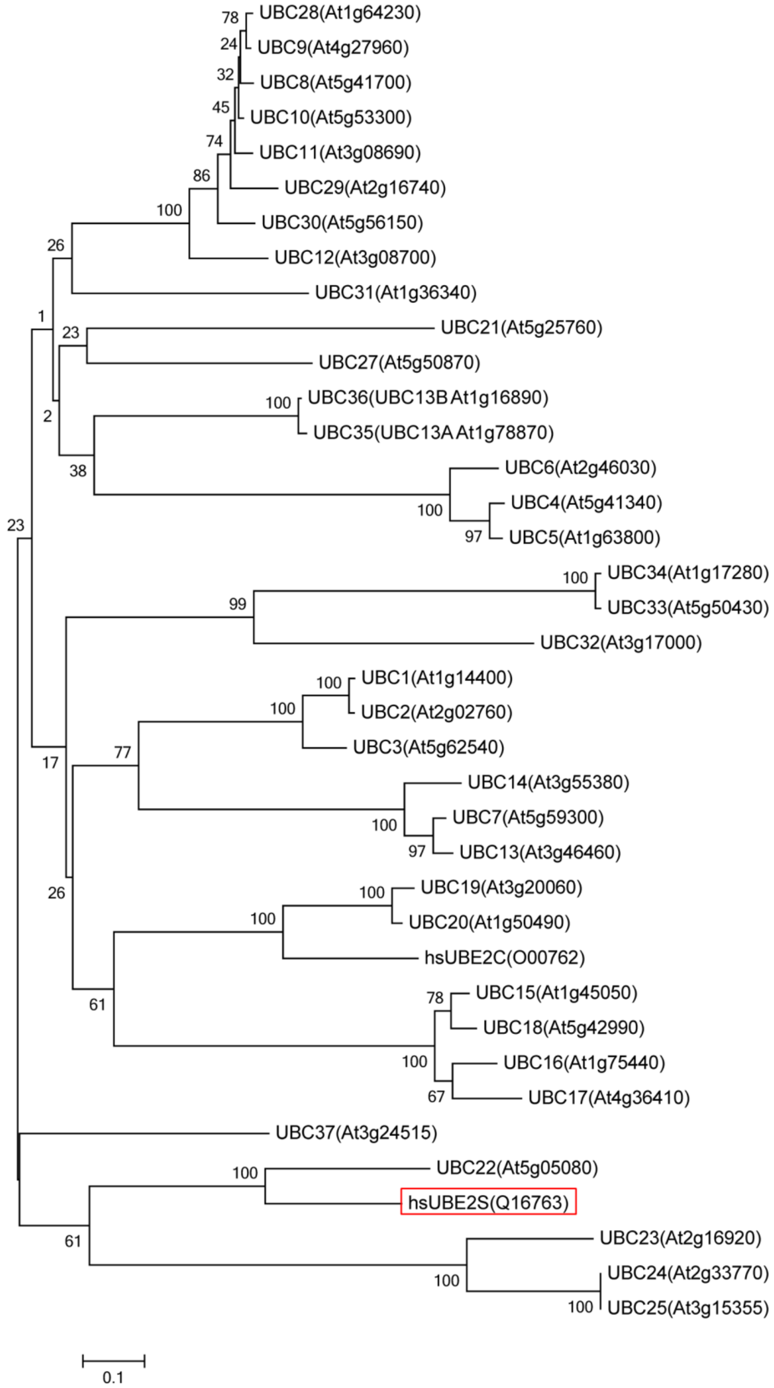

Figure S1. Phylogenetic analysis of Arabidopsis E2s. Thirty six Arabidopsis E2 based on the analysis by (Kraft et al., 2005) were used, except for UBC26 which has no protein sequence data. Two human E2s, UBE2S and UBE2C were also included in the analysis. All the Arabidopsis E2 proteins are listed by their accession numbers and common names. The alignment was made by ClustalX and phylogenetic tree created by Mega 5.1. Bootstrap values from 500 replications for each branch are shown.

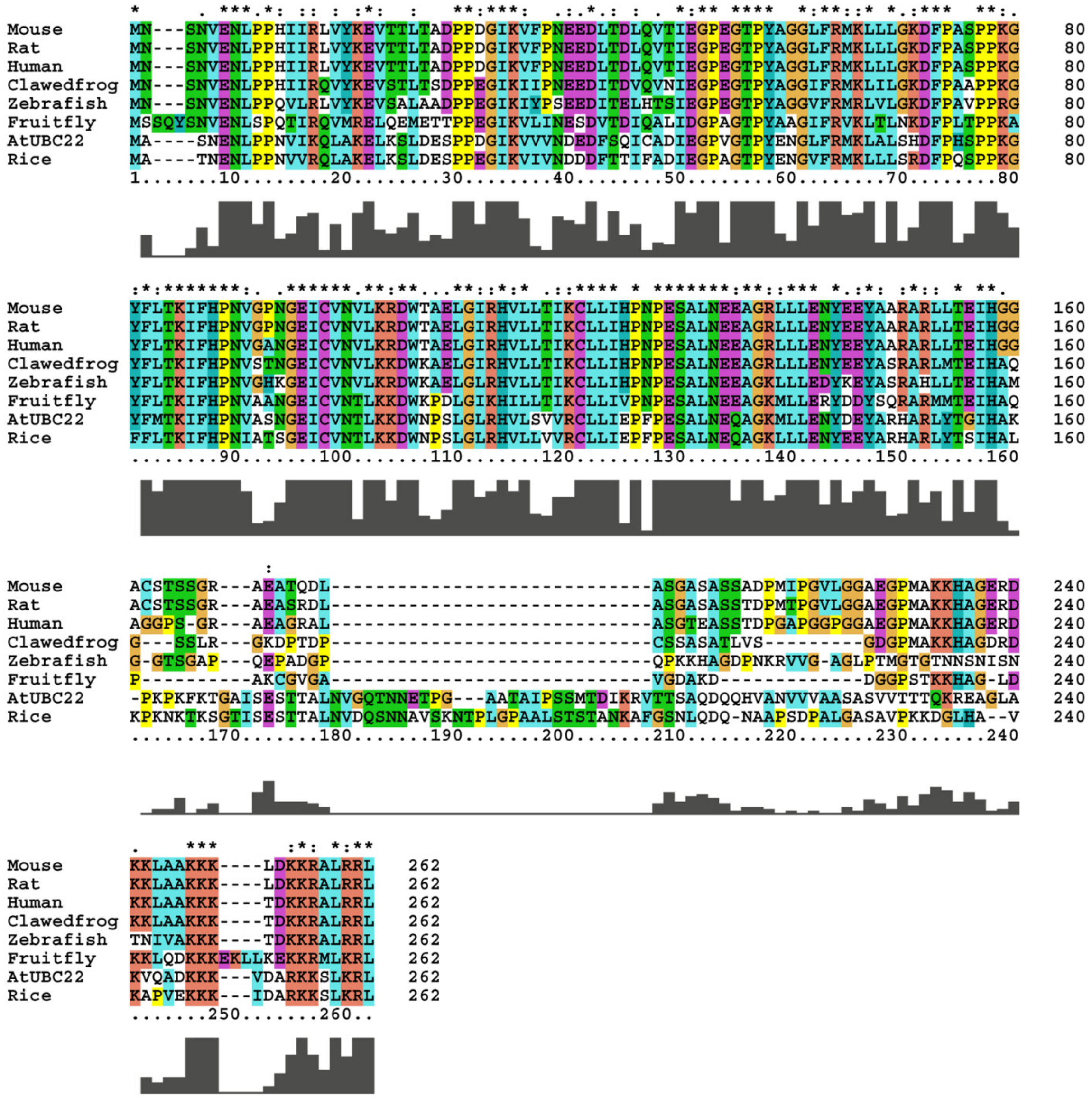

Figure S2. Alignment of putative UBC22 homologues from different species. Alignment was created using ClustalX using default settings. The sequences used are: Mouse, Q921J4; Rat, B5DFI8; Human, Q16763; Clawed frog, Q28F89; Zebrafish, Q4V908; Fruit fly, Q9VX25; Arabidopsis, At5g05080 (UBC22); Rice, Os06g0660700.

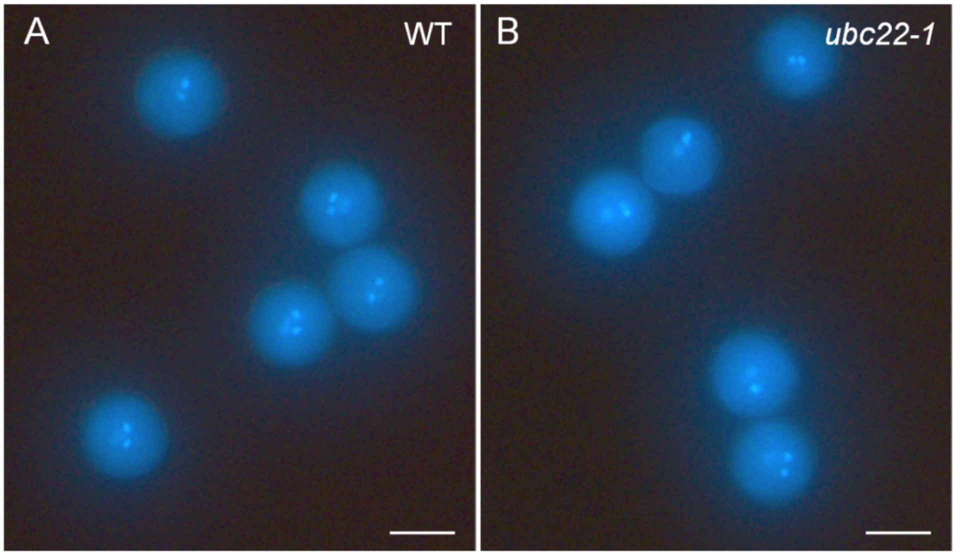

Figure S3. Pollen of WT and *ubc22* mutants. Pollen grains from WT (left) and *ubc22-1* mutants (Right) were stained with DAPI and observed under a fluorescence microscope. Scale bar, 10 μm.

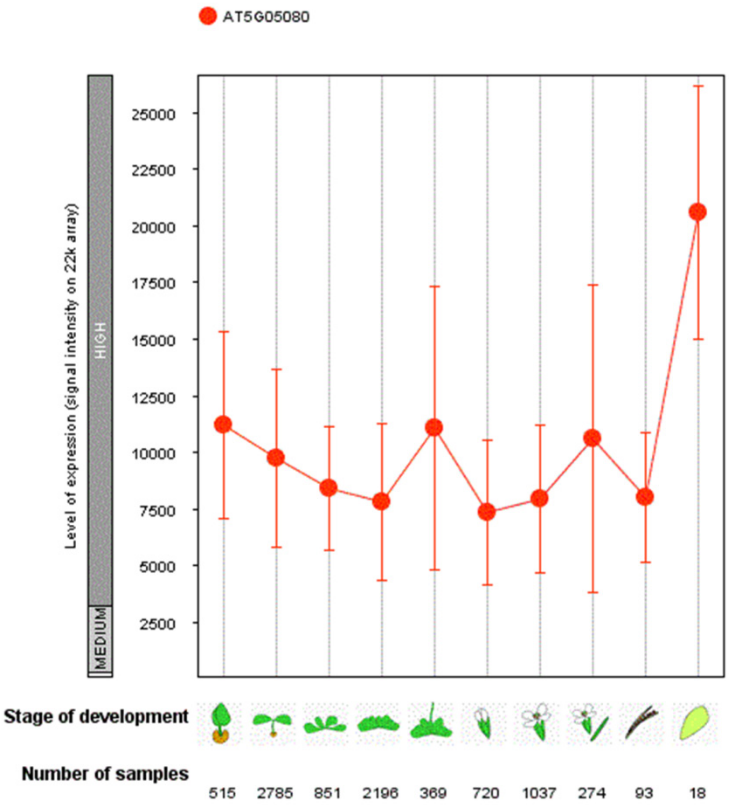

Figure S4. Developmental expression profile of *UBC22*. The figure based on microarray expression data was generated by Genevestigator (https://genevestigator.com). Developmental stages and sample numbers are indicated at the bottom of the figure.

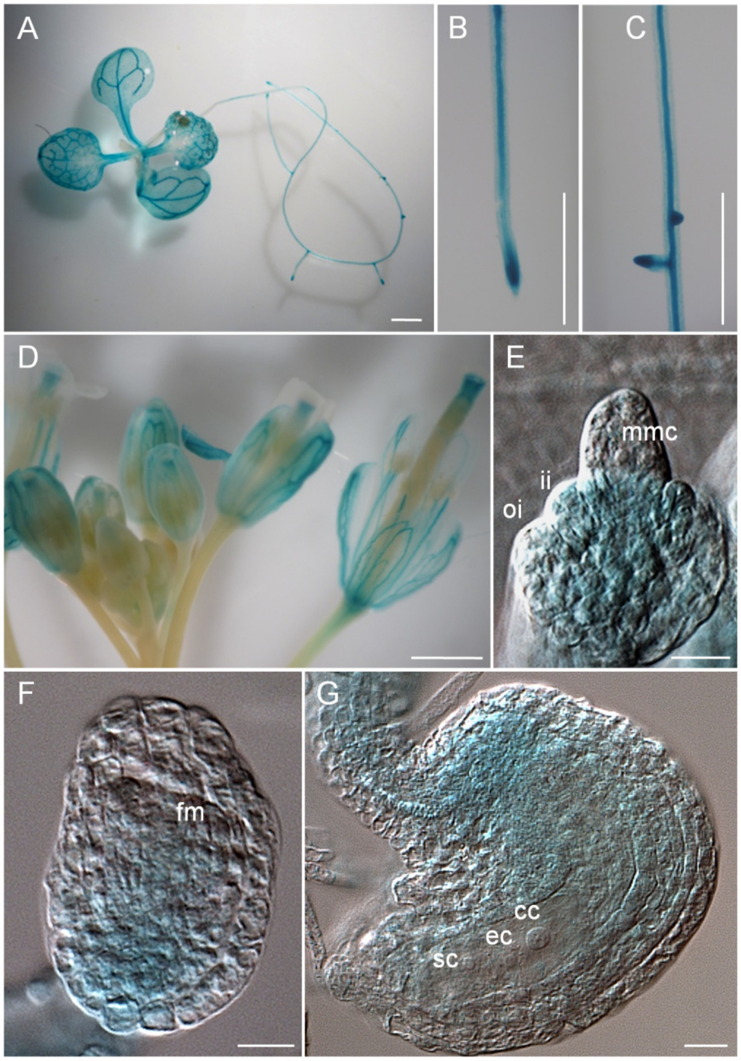

Figure S5. GUS staining of transgenic *Pro$_{UBC22}$:: GUS* plants. Seedling or different tissues from transgenic *Pro$_{UBC22}$:: GUS* plants were analyzed by histochemical GUS staining. Similar patterns were observed in independent transgenic lines although the intensity may vary among the lines. Images from representative plants are shown here. (A) A 10-day old seedling. (B) Root tip and (C) lateral root primordia from a 10-day old seedling. (D) Flower organs from a 6-week old plant. Scale bar, 1 mm. (E, F & G) Ovules at megaspore mother cell (E), functional megaspore (F) and FG7 (G) stages. Abbreviations: mmc, megaspore mother cell; ii, inner integument; oi, outer integument; fm, functional megaspore; cc, central cell; ec, egg cell; sc, synergid cell. Scale bar, 10 µm.

Table S1. Analysis of pollen development in WT and *ubc22-1* mutant plants.

Line	Total pollen counted	Vegetative cell + sperm cells				
		1+2	0+2	1+1	0+1	1+0
WT	1315 (100%)	1208 (91.86%)	86 (6.54%)	21 (1.60%)		
ubc22-1	1761 (100%)	1627 (92.39%)	85 (4.83%)	40 (2.27%)	2 (0.11%)	7 (0.40%)

Table S2. Sequences of primers used for confirming *ubc22* mutants and transcript level of *UBC22* in the complementation lines

Gene name	Primers sequences
UBC22	HW1089: CAG TGT CGA CAA TGG CTA GTA ATG AGA A
(At5g05080)	HW1090: CAG TGC GGC CGC TCA TAG TCT CTT CAA G
	SW17: TGG TTC ACG TAG TGG GCC ATC G
	HW659: ATA TTG ACC ATC ATA CTC ATT GC
At4g33380	HW471: ATG AGA AGC TGG AGG AAG C
	HW472: TCA AGC CGT TAC AAC ACC

REFERENCE

Kraft E, et al. Genome analysis and functional characterization of the E2 and RING-type E3 ligase ubiquitination enzymes of Arabidopsis. Plant Physiol (2005) 139:1597-1611.
